# Effects of Salinity on Tagetes Growth, Physiology, and Shelf Life of Edible Flowers Stored in Passive Modified Atmosphere Packaging or Treated With Ethanol

**DOI:** 10.3389/fpls.2018.01765

**Published:** 2018-12-10

**Authors:** Antonios Chrysargyris, Andreas Tzionis, Panayiota Xylia, Nikos Tzortzakis

**Affiliations:** Department of Agricultural Sciences, Biotechnology and Food Science, Cyprus University of Technology, Limassol, Cyprus

**Keywords:** edible flowers, tagetes, *Tagetes patula*, antioxidant capacity, shelf-life, hydroponics, nutraceutical foods

## Abstract

Irrigation with saline water causes significant crop yield loss. However, short-term saline application might cause less negative effects on yield yet at the same time improve quality aspects of edible products. Tagetes (*Tagetes patula* L.) plants were subjected to salinity (0, 50, and 100 mM NaCl) and harvested flowers were stored up to 14 days in passive modified atmosphere packaging (with or without ethanol application). Salinity of 100 mM NaCl decreased plant biomass and plant size (i.e., height) and had a negative effect on physiological processes such as stomatal closure and chlorophylls content decrease. Salinity increased flower polyphenols, antioxidant activities, and total carotenoids but decreased anthocyanins, and greater impacts were found at salinity of 100 mM NaCl, providing higher antioxidant value of the edible flowers. Short-term saline exposure of tagetes plants activated metabolic processes and as a result there was an accumulation of minerals such as N, P, Na, and Zn on edible flowers. During storage, salinity maintained but ethanol application increased the flower CO_2_ production. Ethanol application decreased the decay of flowers subjected to 100 mM NaCl. Flower weight losses and marketability accelerated at salinity of 100 mM NaCl after 14 days of storage. Tagetes flowers demonstrated induction in both non-enzymatic (i.e., proline content) and enzymatic mechanisms (catalase) to overcome stress caused by salinity during harvest stage and/or ethanol at storage. Our results have shown that short-term exposure to salinity and/or ethanol is able to achieve higher carotenoids and anthocyanins levels and these compounds can be considered as a new source of nutraceuticals.

## Introduction

High consumer preferences in fresh produce with increased popularity of edible flowers is resulting from their important properties for human health because of their abundance in bioactive and nutraceutical components, which offers further marketing opportunities (Mlcek and Rop, 2011). Among others, several plant species are used for disease treatment practices as well as natural additives in foods (Kaisoon et al., 2012; Rachuonyo et al., 2016). The nutritional value of edible flowers is quite similar to the one of leafy vegetables in terms of proteins, fats, polysaccharides, minerals, and vitamins (Upadhyay, 2011) while their antioxidative properties are well appreciated as they are rich in carotenoids and flavonoids (Mato et al., 2000; Friedman et al., 2010).

Intensive cultivation of plants for the production of edible flowers will contribute to the market needs and consumers’ expectations. However, less fertile fields, lack of knowledge about cultivation practices and/or transportation/storage parameters may limit the expansion of edible flowers market. Moreover, salinized land areas are expanding over time along the seaside areas, such as the Mediterranean basin. Salinity is one of the main abiotic factors that decrease crop yields and plant growth by causing hyperionic and hyperosmotic effects on soil solution around rhizosphere (Munns, 2002; Chrysargyris et al., 2018). This results in disturbance of water and minerals uptake by the roots and consequently decrease in yield and quality of the products. Fresh products of lower quality will reflect decrease in storage life of the fresh commodity. As a consequence, producers often have to cope with salinity due to the absence of good quality watering and they have to grow plants in soil or in soilless culture under saline conditions which is of challenge, and occasionally one-way direction. Plants grown in saline environment subjected to physiological and biochemical changes, manage the production of reactive oxygen species (ROS), by activating antioxidative mechanisms. To overcome oxidative stress, plants detoxify ROS by increasing the specific activity of antioxidant enzymes (superoxide dismutase-SOD, catalase-CAT, peroxidase-POX, glutathione peroxidase-GPX, glutathione *S*-transferases-GST, ascorbate peroxidase-APX, dehydroascorbate reductase-DHAR, glutathione reductase-GR, and monodehydroascorbate reductase-MDHAR) or producing non-enzymatic antioxidant molecules (ascorbate, glutathione, α-tocopherol, etc.) (Chrysargyris et al., 2018). The first line for ROS detoxification initiated by SOD increases in order to convert O_2_^-^ to H_2_O_2_, and thereafter the H_2_O_2_ produced is scavenged by catalase and a variety of peroxidases (Tarchoune et al., 2010). Catalase dismutates H_2_O_2_ into H_2_O and O_2_, whereas POX decomposes H_2_O_2_ by oxidation of co-substrates such as phenolic compounds and/or antioxidants (Chrysargyris et al., 2018).

Edible flowers are present in culinary arts by adding flavor, freshness, color, exotic and spicy aroma, improving appearance and are increasingly favored in gourmet cuisine (Kelley et al., 2003). Diverse use of edible flowers is noticed in restaurants and catering services since they are used as garnishes and/or trimming to meals or additives in soups, fresh salads, sweets and savory dishes (Mlcek and Rop, 2011). On top of the fresh use of edible flowers, they can also be consumed dried in drink preparation, in ice cubes in cocktail making and canned in sugar (Mlcek and Rop, 2011).

Like fruit and vegetables, harvested flowers deteriorate quite fast and need to be cooled and stored quickly at chilled temperature (1–5°C) for 2–14 days (Kelley et al., 2003). However, edible flowers could be much more perishable compared to certain fruits. The flowers mainly are highly rotten and their storage duration has a substantial role in determining their retailing value. Fresh produce, including edible flowers, deteriorates with symptoms of browning, chlorophyll bleaching, tissue breakdown, off-flavors and decay. These changes are related to senescence whereas increased rates of respiration, water loss, enzyme activities and/or opportunistic microorganisms’ infection are the key factors for tissue breakdown (Ragaert et al., 2007). The quality and storage duration of edible flowers are firmly associated with the preharvest culturing management used by the producers, including fertigation practices and saline levels. Additionally, postharvest preservation management used in packing houses also influences the quality and storage of fresh commodities, including edible flowers. Among others, ethanol application during postharvest preservation of fresh produce (Han et al., 2006; Tzortzakis and Economakis, 2007; Tzortzakis, 2010) and cut flower (Kaur and Mukherjee, 2013; Begri et al., 2014) has already been examined, while the ethanol application method usually includes dipping rather than vaporization (Begri et al., 2014). The effectiveness of ethanol is related to the increase of the vase life of carnation flowers by preventing the biosynthesis and action of ethylene (Heins and Blakely, 1992).

*Tagetes patula*, commonly known as tagetes/marigold, has various (orange, yellow, mixed) color flowers and bitterish, clove-like flavor (Mlcek and Rop, 2011). Tagetes species are widely known for their flavonoids and terpenes content (Munhoz et al., 2014). As a result, they possess antimicrobial (Gakuubi et al., 2016), insecticidal (Perich et al., 1995), larvicidal (Giarratana et al., 2017), and antioxidant (Fu and Mao, 2008) properties and are used in various countries as traditional medicines to treat colic, diarrhea, vomit, fever, skin diseases and hepatic disorders (Jain et al., 2012).

Not much information is known about the physiology, biochemistry, and postharvest performance of this species when grown in saline environments. The objectives of the present study were to examine (i) the effects of saline levels on tagetes growth, plant physiology and quality of edible flowers, (ii) the postharvest performance of edible flowers from plants grown in saline environments, (iii) the effects of ethanol-treated flowers during chilled storage, and (iv) the combined effect of salinity (in preharvest) and the ethanol application (in postharvest). As a result, this can achieve a better understanding of the responses of tagetes plants to salinity during growth and storage.

## Materials and Methods

### Plant and Experimental Conditions

The present study took place at the greenhouse hydroponic infrastructure of Cyprus University of Technology during the autumn of 2017. Air temperature varied from 29 ± 2°C and 21 ± 2°C during day and night, respectively.

Tagetes (*T. patula* L.) seedlings were grown in nursery (*T* = 18.5–18.8°C; RH = 72–76%, Light:Dark = 16:8 h) for a 3-week period, fertigated with nutrient solution (20-20-20) of electrical conductivity (EC) 2.1 mS/cm for 1 week in order to get plant growth uniformity at the stage of two-true leaves. Seedlings were transplanted in 400 L capacity tanks (40 plants per tank).

### Experimental Set Up

Once tagetes plants were acclimated to the soilless culture environment, they were grown for 42 days to a complete nutrient solution (NS) in deep flow technique (DFT) system. The nutrient solution was refilled with the stock solution every week, to restock nutrients that might have been absorbed. The composition of the stock solution (1:100 v/v) in water was: NO_3_^-^-N = 13.65, K = 7.05, PO_4_-P = 1.29, Ca = 7.63, Mg = 2.81, SO_4_^-2^-S = 1.12, and Na = 1.92 mmol/L, respectively; and B = 30.00, Fe = 25.00, Mn = 10.23, Cu = 0.75, Zn = 4.00, and Mo = 0.51 μmol/L, respectively. The optimal pH and EC of the NS were 5.9 and 2.0 mS/cm, respectively. The NS pH was recorded every other day and tailored accordingly (because of water alkalinity) using H_2_SO_4_ (5% v/v). Nutrient solution was oxygenated twice a day for 0.5 h by means of a pressure pump.

Thereafter, plants were exposed for additional 10 days at three saline (0, 50, and 100 mM NaCl) levels. Each salinity treatment was divided into two tanks and each tank supported four polyethylene trays of 10 plants capacity per tray. The half plants (40 out of 80 plants) were considered further as experimental for the present study. Each saline treatment consisted of four biological replications (10 plants/replication; 40 plants in total for each treatment) which were subjected to further measurements. The regular EC of the nutrient solution was 2.0 mS/cm for the control treatment (0 mM NaCl), 6.0 mS/cm for the salinity of 50 mM NaCl, and 10.0 mS/cm for the salinity of 100 mM NaCl, as it was kept constant during the salinity stress period (Supplementary Figure S1).

### Plant Growth Parameters

One hundred and twenty plants of tagetes were used in the present study. After 6 weeks of plant growth under complete NS plus 10 days of saline short-time stress, four biological replications (each replication was a pool of three measurements in individual plants) for each treatment were studied in detail for plant growth measurements. Plant height, fresh and dry plant weight were measured for the aerial part of the plant (i.e., leaves and stems). The dry matter content was acquired by drying samples to constant weight using a thermo-ventilated oven at 65°C.

### Physiological Parameters and Photosynthetic Pigment Content

Every 2 days for a period of 10 days of saline stress, maximum *F*_v_/*F*_m_ photochemical quantum yields of PSII were determined with an OptiSci OS-30p Chlorophyll Fluorometer (Opti-Sciences) according to Chrysargyris et al. (2017). Chlorophylls (Chl a, Chl b, and total-Chl) content was determined at the end of the experiment based on the method previously described (Chrysargyris et al., 2017). Leaf stomatal conductance was measured on the 4th and 5th sun-exposed leaf from the top of the plant (three measurements per leaf) by using a ΔT-Porometer AP4 (Delta-T Devices-Cambridge, United Kingdom) in accordance with the manufacturer’s instructions.

### Plant Mineral Content

At the end of the experiment, leaf and flower minerals were determined as described in Chrysargyris et al. (2018). Sub samples (0.2–0.3 g) were digested using hydrochloric acid (2 N HCl). K and Na were determined by means of flame photometry (JENWAY, PEP-7 Jenway, Dunmow, United Kingdom), P was determined spectrophotometrically (Multiskan GO, Thermo Fischer Scientific, United States), Mg, Ca, Cu, Fe, and Zn, were determined by an atomic absorption spectrophotometer (PG Instruments AA500FG, Leicestershire, United Kingdom) and N with the help of the Kjeldahl method (BUCHI, Digest automat K-439 and Distillation Kjelflex K-360, Switzerland). Data was expressed in g/kg and mg/kg of dry weight for macro- and micronutrient, respectively.

### Polyphenols and Antioxidant Activity of Leaves and Flowers

Leaves and flowers (0.5 g) polyphenols were extracted (Chrysargyris et al., 2016) with 10 mL of methanol (50% v/v) and supernatant was analyzed for total phenolics and total antioxidant activity by means of the 2,2-diphenyl-1-picrylhydrazyl (DPPH), ferric reducing antioxidant power (FRAP) and 2,2′-azino-bis(3-ethylbenzothiazoline-6-sulfonic acid) (ABTS) methods. Polyphenols content was determined using the Folin-Ciocalteu method at 755 nm according to Marinou et al. (2013) and the results were expressed as equivalents of gallic acid (Scharlau, Spain) per gram of fresh weight. The antioxidant capacity measurement using the DPPH, FRAP, and ABTS assays was performed as previously described (Wojdylo et al., 2007; Chrysargyris et al., 2017). The results for antioxidant activities were expressed in equivalents of trolox per gram of fresh weight.

### Carotenoids and Anthocyanins Content of Flowers

Total carotenoid content was measured at 480 nm as described by Rafiq et al. (2008) with adjustments. Carotenoids were measured using the following equation: Carotenoids (μg) = 4 × A_480_ × volume (mL) (Pessarakli, 1997) and results were expressed as mg of carotenoids per gram of fresh weight.

Total anthocyanins were measured with the pH-differential method (Loizzo et al., 2015) with slight adjustments. In brief, 1 mL of the extract [500 mg in 15 mL methanol/dH_2_O/HCl (70:29:1)] was mixed with (a) 3.5 mL of potassium chloride buffer (0.025 M, pH 1) or (b) 3.5 mL of sodium acetate buffer (0.025 M, pH 4.5). The absorbance of each solution was measured at 520 and 700 nm. The absorbance difference was calculated as follows: *A* = [(*A*_520_ -*A*_700_)_pH_1.0 - (*A*_520_ - *A*_700_)_pH4.5_] and the anthocyanin content was calculated with the following equation: mg of cyanidin 3-glucoside equivalents/L = (absorbance × MW × dilution factor × 1000)/(𝜀 × 1); using the molar absorptivity (𝜀) and molecular weight (MW) of cyanidin 3-glucoside (𝜀 = 26900; MW = 449.2). Results were expressed in mg of cyanidin 3-glucoside equivalents per gram of fresh weight.

### Hydrogen Peroxide Content and Lipid Peroxidation of Flowers

Hydrogen peroxide (H_2_O_2_) content of flowers was determined according to the method of Loreto and Velikova (2001). Flower tissue (0.2 g) was ground in ice cold 0.1% trichloroacetic acid (TCA) and centrifuged at 15000 *g* for 15 min. Aliquot (0.5 mL) of the supernatant was mixed with 0.5 mL of 10 mM potassium-phosphate buffer (pH = 7.0) and 1 mL of 1 M potassium iodide. The absorbance of samples and standards was measured at 390 nm and results were expressed as μmol H_2_O_2_/g fresh weight.

Lipid peroxidation was assessed according to Azevedo-Neto et al. (2006) and measured in terms of malondialdehyde content (MDA). Flower tissue (0.2 g) was homogenized in 0.1% TCA and the extract was centrifuged at 15000 *g* for 10 min. The reaction mixture of 0.5 mL extract and 1.5 mL of 0.5% thiobarbituric acid (TBA) in 20% TCA was incubated at 95°C for 25 min and then cooled on ice bath. The absorbance was determined at 532 nm. Results were expressed as nmol of MDA/g fresh weight.

### Proline Content and Antioxidant Enzymes Activities of Flowers

Harvested flowers (four replicates/treatment) were homogenized in a 50 mM potassium-phosphate ice-cold extraction buffer containing 1 mM ethylenediaminetetraacetic acid (EDTA), 1% (w/v) polyvinylpyrrolidone (PVPP), 1 mM phenylmethylsulfonyl fluoride (PMSF) and 0.05% Triton X-100 (pH = 7.0). Protein content was determined using bovine serum albumin as described previously (Chrysargyris et al., 2017).

Proline was determined according to acid-ninhydrin and toluene method at 520 nm (Khedr et al., 2003). Results were expressed in micrograms of proline per gram of fresh weight. Catalase (CAT) and superoxide dismutase (SOD) activity were assayed according to Jiang and Zhang (2002). CAT was assayed in a reaction mixture (1.5 mL) containing 50 mM K-phosphate buffer (pH 7.0), 10 mM H_2_O_2_ and an enzyme aliquot. The reduction of H_2_O_2_ was measured at 240 nm. The results were expressed in CAT units/mg of protein (1 unit = 1 mM of H_2_O_2_ reduction per min). SOD was assayed using a photochemical method; a reaction mixture (1.5 mL) containing 50 mM K-phosphate buffer (pH 7.5), 13 mM methionine, 75 μM nitro blue tetrazolium (NBT), 0.1 mM EDTA, 2 μM riboflavin and an enzyme aliquot. The reaction began by exposing the mixture to a light source of two 15 watt fluorescent lamps for 15 min and was stopped by placing the tubes in the dark. Absorbance was determined at 560 nm and activity was expressed in units/mg of protein. Peroxidase activity (POD) was determined as described by Tarchoune et al. (2012) following the increase in absorbance at 430 nm. Calculations were performed using the coefficient of extinction of 2.47 mM/cm. One POD unit was defined as the amount of enzyme to decompose 1 μmol of H_2_O_2_ per minute. Results were expressed in units/mg of protein. The activity of APX was determined according to Zhu et al. (2004) by the decrease in the absorbance of ascorbate at 290 nm. Results were expressed in units APX/mg of protein.

### Edible Flower Processing and Shelf-Life Parameters

#### Postharvest Experimental Set Up for Flowers

Harvested plants with flowers were transferred to the laboratory within 30 min and were chosen on the basis of similar flower quality. In order to examine the impacts of salinity on flowers quality, a batch of flowers (5 flowers; ∼38.2 g total weight) was stored directly in polyethylene terephthalate (PET) plastic trays (1 L capacity) up to 14 days. Another batch of flowers was vaporized once with absolute ethanol (0.1% v/v) during a storage of 14 days. The flowers were placed in PET trays (∼4–5 flowers per tray) with snap-on lids, sealed with parafilm and cooled at 5°C, under passive modified atmospheric packaging (MAP). Moisturized (autoclaved dH_2_O) filter paper was placed in each container to maintain high relative humidity during the storage period as described previously (Tzortzakis and Economakis, 2007).

The storage life of fresh produce that maintains not only appearance and safety but also nutritional value is reflected in good quality produce and consumer acceptance (Delaquis et al., 1999). For this reason, after 7 and 14 days of storage at 5°C, CO_2_ concentration inside the plastic trays and quality attributes were recorded on four replications per treatment. Temperature (T°C) and relative humidity (RH %) of the refrigerated trays were monitored during storage period (T 5 ± 0.3°C and RH 87 ± 2%).

#### Determination of CO_2_, Weight Loss, and Color of Flowers

Flower respiration was estimated by measuring CO_2_ concentration of the packages using the Dual gas analyzer (International Control Analyzer Ltd., United Kingdom). Gaseous samples were drawn through septa with a syringe to prevent gas leakage from the packages. Flower weight loss was measured, as the weight of each container was registered before and after storage at 5°C for 7 and 14 days, and results were calculated in weight loss percentage.

The color of the flowers was evaluated with a colorimeter (Chroma meter CR400 Konica Minolta, Japan) where the *L^∗^* (lightness), *a^∗^* (green to red), and *b^∗^* (blue to yellow) values were recorded on day 0, 7, and 14 (three measurements per replicate/four replicates per treatment). The chroma value (C) was calculated with the following equations C = (*a^∗^*^2^+*b^∗^*^2^)^1/2^ (Bolin and Huxsoll, 1991).

#### Determination of Phenolics, Antioxidants, Proline, and Lipid Peroxidation of Flowers

Total phenolic content, carotenoids and anthocyanins content, the DPPH, FRAP, and ABTS scavenging activity, proline content, hydrogen peroxide production, lipid peroxidation, and antioxidant enzymes of flowers were determined as described above following 7 and 14 days of storage.

#### Determination of Decay and Marketability of Flowers

The decay severity was macroscopically evaluated after 14 d of storage with or without ethanol vapor at 5°C/90% RH. The degree of flower decay was rated (at 0.5 intervals) using a scale of 1–4, where 1-clean, no decay, 2-decay less than 25% of the surface, 3-moderate decay (25–50% decay), and 4-severe decay (>50% decay). Plant marketability was also assessed on a 9-point scale (1-low or poor and 9-high or very excellent).

### Statistical Methods

Data was statistically analyzed with the use of IBM SPSS v.21 (IBM Corp., Armonk, NY, United States) software, subjected to analysis of variance (ANOVA), and was expressed by means ± SE (*n* = 4; each replicate consisted of three individual measurements from a pool of plants). Significant differences between mean values were determined using the Duncan Multiple Range Test at *P* = 0.05.

## Results

### Growth and Physiological Parameters

Table 1 presents the effects of salinity concentration into the NS on the plant growth and physiology related parameters. Plant height and leaf stomatal conductance significantly (*P < 0.05*) decreased with salinity of 100 mM NaCl while no differences were found among control and low salinity levels (50 mM NaCl). Both fresh and dry weight of the plants decreased in salinity of 100 mM NaCl. Maximum quantum efficiency of PSII significantly decreased after 3 days of salinity (50 and 100 mM NaCl) stress, whereas it remained unaffected up to the 10th day of saline treatments (Supplementary Figure S2). Chlorophylls (Chl a and total Chls) decreased with the application of 100 mM NaCl (Table 1). No differences were found for leaf total phenolics (averaged in 18.77 mg GAE/g Fw) and antioxidant activity (averaged in 7.48, 4.46, and 2.29 mg trolox/g Fw for FRAP, DPPH, and ABTS, respectively) between control and saline plants (data not shown).

**Table 1:** Effect of salinity levels (0, 50, and 100 mM NaCl) on tagetes plant height (cm), biomass fresh and dry weight (Fw, Dw; g/plant), leaf stomatal conductance (mmol/m^2^/s), leaf Chlorophyll a (Chla; mg/g Fw), Chlorophyll b (Chlb; mg/g Fw), total Chlorophylls (total Chl; mg/g Fw) in plants grown hydroponically.

	0 mM NaCl	50 mM NaCl	100 mM NaCl
Plant height	35.00 ± 0.73a^Y^	35.16 ± 1.42a	30.66 ± 1.43b
Biomass Fw	98.02 ± 8.31ab	122.75 ± 18.39a	80.96 ± 6.91b
Biomass Dw	11.23 ± 0.69ab	14.56 ± 2.04a	10.41 ± 0.76b
Stomatal conductance	412.50 ± 19.84a	396.66 ± 37.23a	184.33 ± 31.10b
Chlorophyll a	1.73 ± 0.03a	1.74 ± 0.05a	1.39 ± 0.02b
Chlorophyll b	0.77 ± 0.05a	0.61 ± 0.01b	0.42 ± 0.01c
Chlorophyll total	2.50 ± 0.07a	2.36 ± 0.05a	1.82 ± 0.01b


*^*Y*^Results are expressed as means ± SE (*n* = 4). Values in rows followed by the same letter are not significantly different, *P* ≤ 0.05.*

Considering the flowers produced by tagetes plants, salinity treatment increased several flowers physiological parameters tested in this study (Table 2). Total phenolics and carotenoids as well as antioxidant activities (determined by FRAP, DPPH, and ABTS methods) increased with the application of salinity, especially at the high levels of 100 mM NaCl. The opposite was evident concerning the anthocyanins content as salinity decreased the anthocyanins up to 65%, compared with the control treatment (Table 2). No differences were found in flower Chroma, and *L*, *a^∗^*, and *b^∗^* color values (Supplementary Table S1).

**Table 2:** Effect of salinity levels (0, 50, and 100 mM NaCl) on tagetes flowers total phenolics (μmol GAE/g Fw), antioxidant activity (FRAP, DPPH, ABTS: mg trolox/g Fw), carotenoids (mg/g Fw), and anthocyanins (mg cyn-3-glu/g Fw) in plants grown hydroponically.

	0 mM NaCl	50 mM NaCl	100 mM NaCl
Total phenols	50.59 ± 1.58b^Y^	57.20 ± 0.66a	61.60 ± 2.92a
FRAP	40.90 ± 0.64b	53.00 ± 2.46a	47.84 ± 2.74a
DPPH	21.93 ± 0.44b	25.68 ± 1.60ab	28.40 ± 1.77a
ABTS	3.96 ± 0.21b	4.49 ± 0.11a	4.53 ± 0.13b
Carotenoids	0.155 ± 0.0127b	0.212 ± 0.0020a	0.187 ± 0.0107a
Anthocyanins	0.084 ± 0.0132a	0.029 ± 0.0054b	0.038 ± 0.0062b


*^*Y*^Results are expressed as means ± SE (*n* = 4). Values in rows followed by the same letter are not significantly different, *P* ≤ 0.05.*

Considering that H_2_O_2_ production indicates an induction of salinity stress, the greatest production was found in tagetes flowers derived from 100 mM NaCl-stressed plants, followed by the once subjected to salinity of 50 mM NaCl (Table 3). Salinity increased the production of H_2_O_2_ and proline content but decreased the APX activity. Low saline levels of 50 mM NaCl increased the activity of both CAT and POD while high salinity of 100 mM NaCl had similar levels to the control for both enzymes’ activities. No differences were found concerning lipid peroxidation and SOD activities among treatments (Table 3).

**Table 3:** Effect of salinity levels (0, 50, and 100 mM NaCl) on tagetes flowers on the lipid peroxidation (MDA; nmol/g Fw), hydrogen peroxide production (H_2_O_2_; μmol/g Fw), proline content (μg/g Fw), and antioxidant enzymes activities (SOD, CAT, POD, APX in units/mg protein) in plants grown hydroponically.

	0 mM NaCl	50 mM NaCl	100 mM NaCl
H_2_O_2_	17.30 ± 0.07c^Y^	18.63 ± 0.25b	23.30 ± 0.57a
MDA	51.26 ± 1.27a	58.79 ± 0.74a	58.34 ± 3.44a
Proline	0.287 ± 0.007c	1.312 ± 0.030b	1.997 ± 0.026a
SOD	14.02 ± 0.04a	15.72 ± 0.80a	15.09 ± 0.01a
CAT	16.27 ± 0.32b	19.45 ± 0.01a	16.72 ± 0.38b
POD	0.39 ± 0.03b	0.61 ± 0.07a	0.36 ± 0.02b
APX	3.79 ± 0.04a	2.23 ± 0.04b	1.43 ± 0.01c


*^*Y*^Results are expressed as means ± SE (*n* = 4). Values in rows followed by the same letter are not significantly different, *P* ≤ 0.05.*

### Leaf and Flower Mineral Content

Mineral content in leaves and flowers is presented in Table 4, with effects of salinity to be mainly noticed on the flowers rather than the leaves. Therefore, in flowers, increased salinity levels resulted in increased (*P* < 0.05) Na and N content and decreased Fe content. Flowers accumulated more P and Zn when the tagetes plants were subjected to both salinity levels when compared to the control plants. Salinity of 100 mM NaCl decreased (*P* < 0.05) the Mg content in flowers. The levels of K and Cu did not vary among the treatments. Interestingly, salinity of 50 mM NaCl accumulated Ca in flowers almost three times compared to control treatment (non-saline treated plants) (Table 4).

**Table 4:** Effect of salinity levels (0, 50, and 100 mM NaCl) on tagetes leaves and flowers minerals in plants grown hydroponically.

Leaves	0 mM NaCl	50 mM NaCl	100 mM NaCl
N (g/kg)	33.45 ± 1.65aY	33.94 ± 2.38a	29.32 ± 0.87a
K (g/kg)	21.55 ± 1.73a	23.48 ± 0.89a	23.76 ± 0.97a
Ca (g/kg)	4.75 ± 0.61b	11.87 ± 2.17a	13.91 ± 2.93a
P (g/kg)	6.15 ± 0.66a	6.89 ± 0.52a	4.95 ± 0.60a
Mg (g/kg)	4.14 ± 0.65a	3.40 ± 0.67a	4.06 ± 0.52a
Na (g/kg)	4.99 ± 0.45b	5.28 ± 0.51b	9.35 ± 1.12a
Fe (mg/kg)	180.55 ± 14.29a	104.30 ± 1.92b	108.90 ± 6.07b
Zn (mg/kg)	109.78 ± 3.13a	110.71 ± 4.96a	98.68 ± 3.38b
Cu (mg/kg)	38.69 ± 9.22a	46.09 ± 11.39a	47.99 ± 11.44a

**Flowers**	**0 mM NaCl**	**50 mM NaCl**	**100 mM NaCl**

N (g/kg)	15.81 ± 0.33c	17.26 ± 0.14b	19.22 ± 0.18a
K (g/kg)	11.77 ± 0.46a	11.44 ± 0.35a	10.98 ± 0.77a
Ca (g/kg)	4.96 ± 1.76b	12.33 ± 2.24a	3.73 ± 1.55b
P (g/kg)	4.14 ± 0.22b	4.77 ± 0.0.08a	5.03 ± 0.08a
Mg (g/kg)	0.54 ± 0.05a	0.47 ± 0.04a	0.27 ± 0.03b
Na (g/kg)	2.79 ± 0.03c	3.49 ± 0.13b	4.06 ± 0.08a
Fe (mg/kg)	106.16 ± 4.09a	71.03 ± 12.13b	50.54 ± 6.10b
Zn (mg/kg)	76.20 ± 6.81b	110.37 ± 2.54a	116.25 ± 2.24a
Cu (mg/kg)	22.63 ± 8.92a	27.74 ± 6.21a	26.92 ± 6.65a


*^*Y*^Results are expressed as means ± SE (*n* = 4). Values in rows followed by the same letter are not significantly different, *P* ≤ 0.05.*

In the case of tagetes leaves, salinity of 100 mM NaCl accumulated (*P* < 0.05) more Na but less Zn when compared to low (50 mM NaCl) salinity or control treatment (Table 4). Salinity in both low and high levels accumulated Ca in leaves but had lesser Fe content when compared with the non-saline treated plants. No differences were found for N, K, P, Mg, and Cu content among treatments.

### Edible Flower Processing and Shelf-Life Parameters

**FIGURE 1:**
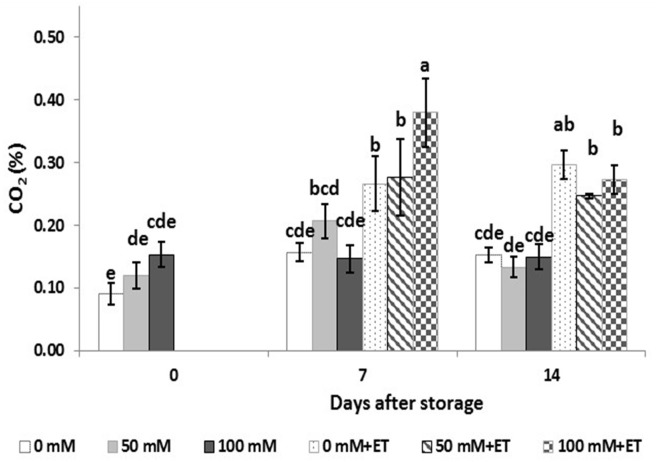
Effect of salinity levels (0-50-100 mM NaCl) on greenhouse-grown tagetes flowers in modified atmosphere packaging (with or without vaporized ethanol-ET) on the respiration of treated flowers during 7 and 14 days of storage at 5°C. Significant differences (*P* < 0.05) among treatments are indicated by different letters. Error bars show SE (*n* = 4).

The levels of CO_2_ accumulated in the head space of saline and/or ethanol processed tagetes edible flowers, which is depending on the respiration rates, varied according to time of storage as well as the treatments applied (Figure 1). Tagetes flowers obtained by saline-treated plants immediately after harvest had no significant differences in CO_2_ concentration inside the trays, despite the increase trend of CO_2_ concentration as the salinity levels were increased. Following storage up to 14 days, salinity did not have any effect on CO_2_ concentration. Interestingly, the vapor application of ethanol increased CO_2_ concentration in trays derived by flowers respiration during storage, compared to untreated ethanol flowers. This increase of flowers respiration was greater in plants grown at 100 mM NaCl at 7 days compared to 50 mM NaCl and control treatments.

No differences were found on flower color indicated as Chroma (ranged from 83.44 to 94.79), *L* (ranged from 48.23 to 56.72), *a^∗^* (ranged from 35.99 to 43.13), and *b^∗^* (ranged from 71.86 to 86.11) color values on flowers during postharvest storage (Supplementary Table S2).

**FIGURE 2:**
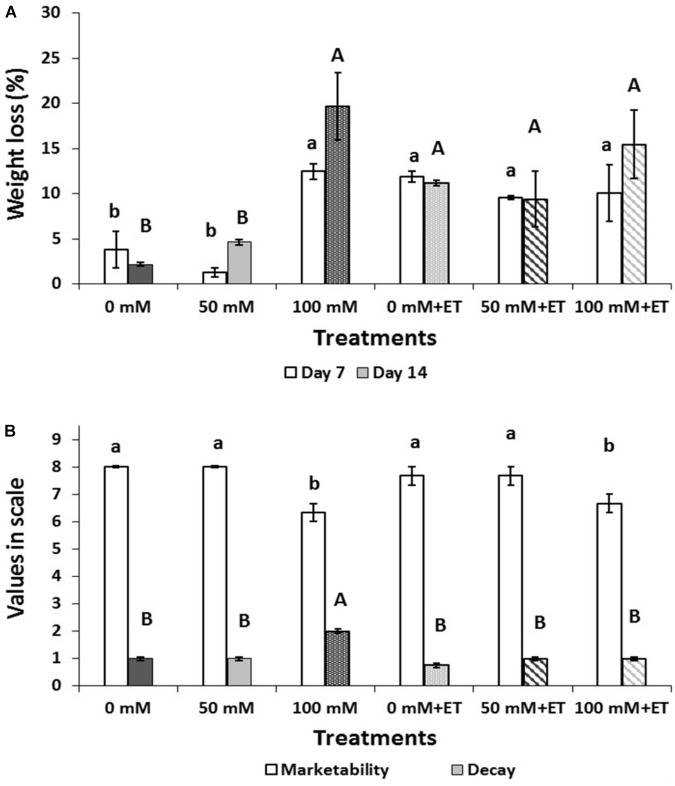
Effect of salinity levels (0-50-100 mM NaCl) on greenhouse-grown tagetes flowers in modified atmosphere packaging (with or without vaporized ethanol-ET) on the **(A)** weight loss and **(B)** marketability (scale 1–9) and decay (scale 1–4) of treated flowers during 7 and 14 days of storage at 5°C. Significant differences (*P* < 0.05) among treatments are indicated by different letters. Error bars show SE (*n* = 4).

Weight loss significantly (*P* < 0.05) increased at salinity of 100 mM NaCl during storage (Figure 2A), as on days 7 and 14 weight loss of tagetes reached 12.48%, and 19.64%, respectively. Ethanol application increased water loss in both control and 50 mM saline-treated flowers (Figure 2A).

Flower marketability and decay after 14 days of storage are presented in Figure 2B. Therefore, the marketability of flowers decreased (*P* < 0.05) when plants were grown at high (100 mM NaCl) saline levels, while ET itself did not change the marketability of the edible flowers. Moderate decay up to 50% was found in 100 mM NaCl-treated flowers. Interestingly enough, ET application alleviated the induced decay found in 100 mM NaCl-treated flowers to levels similar to the relevant control treatment (0 mM NaCl+ET).

**FIGURE 3:**
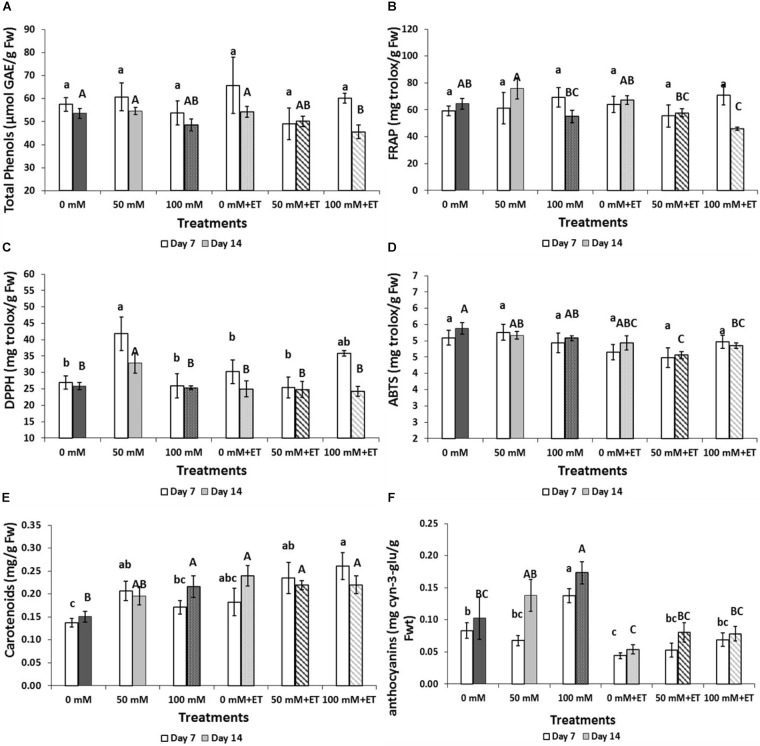
Effect of salinity levels (0-50-100 mM NaCl) on greenhouse-grown tagetes flowers in modified atmosphere packaging (with or without vaporized ethanol-ET) during storage at 5°C on the content of total phenols, carotenoids, anthocyanins, and antioxidant activity. **(A)** Total phenols, **(B)** FRAP, **(C)** DPPH, **(D)** ABTS **(E)** carotenoids, and **(F)** anthocyanins. Significant differences (*P* < 0.05) among treatments are indicated by different small or capital letters for days 7 and 14, respectively. Error bars show SE (*n* = 4).

Total phenolics were reduced in salinity of 100 mM NaCl with ethanol compared to non-saline harvested flowers exposed to ET vapors (0 mM NaCl+ET), following 14 days of storage at 5°C (Figure 3A). Antioxidant activities decreased mainly due to the increased saline levels, storage duration and/or ET vapor as assayed by DPPH, FRAP, and ABTS methods (Figures 3B–D). The content of carotenoids increased due to saline- and ET-application as well as the storage period (7 days versus 14 days of storage) (Figure 3E). Thus, carotenoids increased with the application of low (50 mM NaCl) salinity compared to the control treatment following 7 days of storage. The highest anthocyanins content was found in flowers treated with salinity of 100 mM NaCl and stored for 7 and 14 days (Figure 3F) while ET-vaporized flowers revealed low content of anthocyanins compared to the non-vaporized flowers.

**FIGURE 4:**
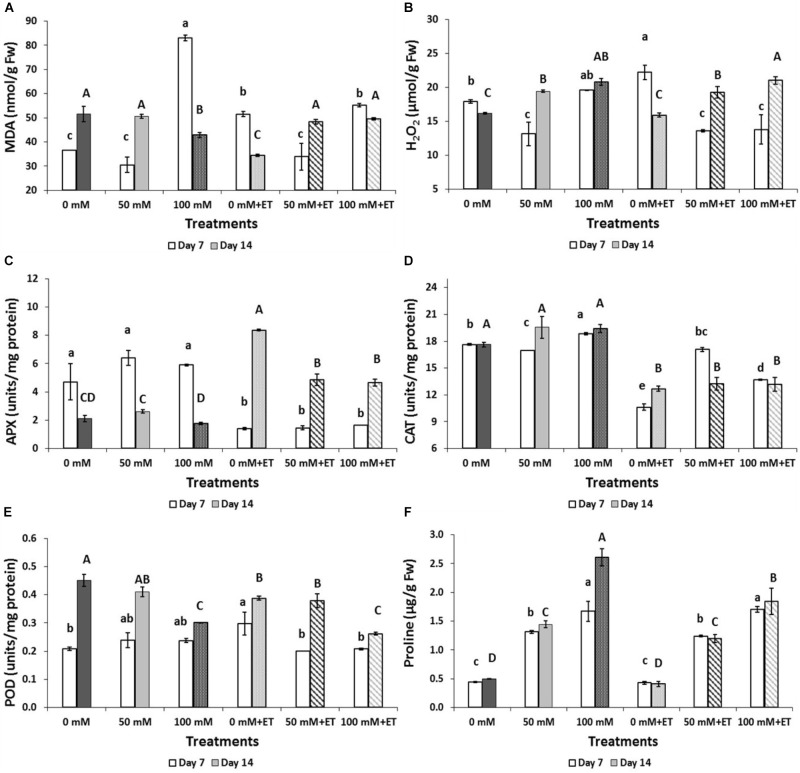
Effect of salinity levels (0-50-100 mM NaCl) on greenhouse-grown tagetes flowers in modified atmosphere packaging (with or without vaporized ethanol-ET) during storage at 5°C on the flower damage index, proline, and antioxidant enzymes activities on greenhouse-grown tagetes. **(A)** Lipid peroxidation (MDA), **(B)** H_2_O_2_, **(C)** APX, **(D)** CAT, **(E)** POD, **(F)** proline. Significant differences (*P* < 0.05) among treatments are indicated by different small or capital letters for Day 7 and 14, respectively. Error bars show SE (*n* = 4).

Salinity of 100 mM NaCl increased lipid peroxidation as measured by MDA concentration after 7 days of storage while ET caused less increase in MDA (Figure 4A). After 14 days of storage, MDA decreased in salinity of 100 mM NaCl or in ET non-saline treated flowers. The production of H_2_O_2_ increased with the application of ET after 7 days of storage (Figure 4B). Indeed, ET combination with salinity (50 mM NaCl+ET and 100 mM NaCl+ET) revealed H_2_O_2_ decreases to similar levels as the 50 mM NaCl.

Following 7 days of storage, salinity of 100 mM NaCl increased the CAT activity and proline content (Figures 4D,F) while no differences were found in APX and POD activities (Figures 4C,E). At 14 days of storage, salinity of 100 mM NaCl decreased POD activity and increased proline content whereas APX and CAT activities remained at similar levels to the control treatment (non-saline flowers) (Figures 4C–F).

ET application, following 7 days of storage, decreased both APX and CAT but increased proline content (Figures 4C,D,F) and this effect persisted till the 14th day for CAT and proline. The application of ET in saline-treated flowers decreased POD and increased CAT and proline content after 7 days of storage while APX was reduced after 14 days of storage with the salinity+ET combination (Figures 4C–F). No differences were found in SOD activity among treatment and/or days of storage (data not shown).

## Discussion

The present study investigated the impacts of saline levels on plant growth and physiology, mineral content and storability of tagetes edible flowers. In addition, ethanol application was tested under passive modified atmospheric packaging as a well-known means of postharvest preservation of fresh produce (Tzortzakis and Economakis, 2007). Cut flowers are the reproductive organs of a plant and they are more sensitive to deterioration and tissue break down than the relevant plant vegetative parts, i.e., leaves and stems (Serek and Reid, 2000). Edible flowers are cut off the stem at the pedicel and, therefore, they are more sensitive and exposed to additional stress. However, edible flowers had previously received less attention than cut flowers and even lesser attention than fresh fruits and vegetables due to their low production and market interest (Kou et al., 2012).

Plants exposed to saline conditions often demonstrate a decrease in the ability to absorb water and minerals causing rapid reduction in growth and yield and inducing several metabolic changes (Hendawy and Khalid, 2005; Chrysargyris et al., 2018). Salt stress is complex and leads to the formation of ROS such as singlet oxygen (O_2_^1^), superoxide (O_2_^-^) and hydrogen peroxide (H_2_O_2_) with harmful effects on biomolecules (lipids, proteins, nucleic acids) due to oxidative damage (Gill and Tuteja, 2010; Chrysargyris et al., 2018). In the current study, 100 mM NaCl significantly (*P* < 0.05) reduced plant biomass and plant size (i.e., height) and affected physiological processes negatively. Therefore, plants closed the leaf stomata to overcome salinity stress and at the same time salinity caused osmotic stress and water deficiency, which was determined by the stomatal conductance reductions. Stomatal conductance reduction is an adaptation plant mechanism to salinity stress and has been reported in *Plandago* spp. (Izadi-Darbandi and Mehdikhani, 2018) and *Calendula* *officinalis* (Khalid and Teixeira da Silva, 2010). The reduction in growth is also related to the decreased content of chlorophylls as observed at 100 mM NaCl application, which can be one of the factors for the photosynthetic rates decrease (Kiarostami et al., 2010). Plant growth and chlorophylls content reductions under saline conditions have been reported for several species (Tarchoune et al., 2010; Chondraki et al., 2012; Klados and Tzortzakis, 2014; Chrysargyris et al., 2018). Chaparzadeh et al. (2004) reported plant biomass reduction in *C.* *officinalis* subjected to salinity of 100 mM NaCl on a long term basis while Villarino and Mattson (2011) observed such reductions at the *T.* *patula*. Tagetes plants subjected to saline conditions exhibited an *F*_v_/*F*_m_ ratio higher than 0.80, indicating absence of severe stress (Bjorkman and Demmig, 1987), as this is related to the short-term exposure to salinity. Indeed, the application of short-term saline stress (10 days) did not change the Chroma and *L*, *a^∗^*, and *b^∗^* color values of the flowers. Therefore, visually flowers maintained their marketability value on chroma related parameters.

Plants cope with salinity induced stress by altering metabolic processes and stimulating the formation of phenolics and antioxidant activity to scavenge free radicals and ions chelators (Balasundram et al., 2006). Examining polyphenols and antioxidant activities in leaves and flowers leads to the conclusion that the effects of salinity could be clearly seen only on the latter (i.e., flowers). Therefore, flowers polyphenols and antioxidant activities (determined by three antioxidant assays of FRAP, DPPH and ABTS) as well as carotenoids increased with the application of salinity, with more pronounced impacts at salinity of 100 mM NaCl. This indicates higher nutraceutical value for the edible flowers when antioxidants and carotenoids increased in flowers subjected to salinity stress. Similar findings were found by Chaparzadeh et al. (2004) at *C.* *officinalis* flowers. Considering that plants change metabolic processes due to salinity stress, changes first take place at the vegetative part (i.e., leaves) and then are followed by changes at the reproductive organs (i.e., flowers). Thus, unchanged polyphenols and antioxidants in leaves possibly indicate that any inductions as a reaction of the plant to overcome saline stress took place before the 10th day. The decrease of anthocyanins in flowers subjected to salinity could be correlated with the decrease in plant growth/development as several metabolic processes were slowed down, other pigments accumulation or that anthocyanins have been already involved at the antioxidative mechanisms of the plant and their content had been exhausted. However, further studies requited to that direction, as the effect of salinity on anthocyanins accumulation is varied. Borghesi et al. (2011) for example, showed an opposite behavior in anthocyanins accumulation in two tomato genotypes when subjected to salinity.

Tagetes flowers were richer in potassium (averaged in 11.40 mg/kg dry weight) than in sodium (averaged in 3.45 mg/kg dry weight), which is quite useful for preventing cardiovascular diseases. Short-term saline exposure of tagetes plants activated metabolic processes such as accumulation of minerals (such as N, P, and Zn) on edible flowers which is of great importance to human health. Zinc deficiency causes impaired growth, immune dysfunction, increased morbidity and mortality as well as abnormal neuro-behavioral development (Mayer et al., 2008). Nitrogen and phosphorus have a substantial role in several metabolic processes since phospholipids, as a constitute of nucleic acid, are involved in protein synthesis, DNA, RNA, and ATP (Rouached et al., 2010). Consumption of 12 g of dry *T. patula* in salads, for example, can provide the 25% of the daily needs of potassium for adults (Rop et al., 2012).

Salinity and/or ET application causes stress which benefits the accumulation of ROS. Plants develop scavenging mechanisms against ROS detoxification by increasing the activity of antioxidant enzymes, such as SOD, APX, CAT, and glutathione reductase (GR) (Foyer and Noctor, 2011). In the present study, tagetes flowers demonstrated activation of both non-enzymatic (i.e., proline content) and enzymatic mechanisms (CAT) to overcome ROS detoxification. High accumulation of proline in plant tissue is an important adaptive mechanism of salt tolerance as proline is regarded as a source of energy, carbon and nitrogen for the recovering tissues, by acting as a compatible solute in osmotic adjustment and reduces membrane oxidative damage (Hossain et al., 2014). Proline acts as an osmolyte and reduces the osmotic potential, thus reducing toxic ion uptake (Chrysargyris et al., 2018). CAT is valuable for the elimination of hydrogen peroxide in peroxisomes by oxidases involved in β-oxidation of fatty acids, photorespiration and purine catabolism (Garg and Manchanda, 2009). The decreased enzymatic activities of APX and POD or the unaffected SOD activity is probably indicating that either enzymes were not involved in H_2_O_2_ scavenging or enzymes activities have been spent for H_2_O_2_ capture and ROS in general at an earlier stage. SOD is used for plant primary detoxification and is later followed by APX and POD (Chrysargyris et al., 2018).

Very few studies have examined the storage conditions of edible flowers and a gap of knowledge is evidenced on the postharvest preservation of edible flowers (Kou et al., 2012). The storage period of edible flowers is usually limited within a couple of days so high-tech storage and shipping conditions are required to extent their shelf life and simultaneously reduce the deteriorated products waste. Moreover, crop cultivation in controlled manner, as soilless culture, can yield produce of high quality and possible storability. Modified atmospheric packaging is commonly used for fresh produce quality maintenance, prolonging shelf life, and decreasing the microbial load of perishable commodities (Tzortzakis, 2010; Kou et al., 2012). Fresh produce stored on MAP conditions usually demonstrates an increase in carbon dioxide and a decrease in oxygen concentration and this actually retards the produce respiration process. In the present study ethanol enhanced the flower metabolic processes as it increased CO_2_ production in tagetes flowers stored for 14 days, while salinity and/or storage period had no significant effects on it. Similarly, Kou et al. (2012) reported increased CO_2_ accumulation in edible carnations and snapdragons following 7 days of storage. Ethanol prevents a rise in respiration rate and autocatalytic ethylene production/action (Asoda et al., 2009).

The increase in CO_2_ production was followed by weight loss in flowers subjected to ET even from the 7 days of storage. Flower weight loss increased (up to two and eightfold) in 100 mM NaCl-treated plants following 7 and 14 days of storage, respectively. This affected the flower marketability as it was decreased (*P* < 0.05) when plants were grown at high (100 mM NaCl) saline levels while ET itself did not change the marketability of the flowers. Therefore, tagetes flowers did not benefit on marketability terms with the application of ET, as it was previous reported on fruits and vegetables, whereas ethanol can be used for fresh produce preservation (Pesis, 2005; Asoda et al., 2009). Even though ethanol did not improve flower marketability, ET application alleviated the induced decay found in 100 mM NaCl-treated flowers to levels similar to the relevant control treatment (0 mM NaCl+ET). The antimicrobial properties of ethanol have been reported in several commodities (Karabulut et al., 2004; Tzortzakis, 2010). The increased decay observed at salinity of 100 mM NaCl-treated flowers may be related to the increased respiration metabolism and weight loss of the flowers but also to the high moisture content inside the tagetes packaging, as strong condensation was evidenced.

During storage, flowers maintained their color as several indicators (Chroma, *a^∗^*, *b^∗^*, *L*) remained at similar levels. Therefore, neither saline levels nor ET-vapors affected the color of tagetes flowers which established the maintenance of marketability, and the possible utilization of salinity and/or ET for improve/maintain the edible flowers quality.

The content of polyphenols decreased in flowers subjected to stress of salinity of 100 mM NaCl with ethanol after 14 days of storage at chilled temperature of 5°C whereas flower antioxidant activity (assayed by DPPH, FRAP, and ABTS) decreased mainly due to increased saline levels, storage duration and/or ET vapor. Thus, flowers were gradually deprived of their ability to detoxify ROS after 14 days of storage and alternative processes such as carotenoids and flavonoids accumulation might have taken place. The stress caused by saline- and ET-application resulted in increased content of carotenoids whereas higher anthocyanins content was found in flowers treated with salinity of 100 mM NaCl and stored for 7 and 14 days. This is of great importance, as stressed flowers by saline and/or ET were able to achieve higher carotenoids and anthocyanins levels and be a new source of nutraceutical foods. El Kereamy et al. (2002) reported that ethanol triggers gene expression leading to accumulation of anthocyanins during berry ripening, when ethanol (5% v/v) was applied on Cabernet Sauvignon grape fruit at veraison stage. The increased consumer and market demand for plant-based products with high antioxidant status may acknowledge edible flowers subjected to short-term salinity stress as products of added value which could prevent oxidative damage in human health. Fresh produce consumption with antioxidants could prevent chronic diseases such as type-2 diabetes, cancer, cardiovascular, and neurodegenerative disorders (Petrova et al., 2016).

## Conclusion

Edible flowers are rich in flavonoids, phenolics, and terpenes, exhibit biocidal activities and possess beneficial properties for human health. Unfortunately, in spite of their agronomic potential, the concept of eating flowers is still viewed with doubt. Considering that salinity maintained the flower quality and storability, despite the reduction in growth and productivity of saline-treated plants, producers can consider the use of saline water for irrigation needs as a short-time stress for tagetes crops. Edible flowers subjected to saline conditions accumulated minerals such as N, P, Na, and Zn. Short-term exposure to saline condition and/or ethanol vapor application trigger flower metabolic process (both non-enzymatic (i.e., proline content) and enzymatic mechanisms (catalase) to overcome stress) and resulted higher carotenoids and anthocyanins levels during storage. It is the activation of several important metabolic processes with higher antioxidant status of the edible flowers that can be considered as a source of nutraceutical foods. Further efforts are needed to improve the postharvest preservation of the flowers when subjected to saline-stress conditions.

## Data Availability

All datasets generated for this study are included in the manuscript and the Supplementary Files.

## Author Contributions

AC, AT, and NT designed and performed the experiments and physiological measurements. AC, PX, and AT performed the postharvest measurements. AC and NT analyzed the data and critically discussed the data. NT and AC prepared the manuscript. NT coordinated the research.

## Conflict of Interest Statement

The authors declare that the research was conducted in the absence of any commercial or financial relationships that could be construed as a potential conflict of interest.

## Supplementary Material

Figure S1 Fluctuation of drainage pH and EC (mS/cm) under different salinity levels (0, 50, and 100 mM NaCl) in hydroponically grown tagetes plants.

Figure S2 Effect of salinity levels (0, 50, and 100 mM NaCl) on tagetes maximum quantum efficiency of PSII (*F*_v_/*F*_m_) in plants grown hydroponically.

Table S1 Effect of salinity levels (0, 50, and 100 mM NaCl) on tagetes flowers color values (*L*, *a^∗^*, *b^∗^*, Chroma) in plants grown hydroponically. ^*Y*^Results are expressed as means±SE (*n* = 6). Values in rows followed by the same letter are not significantly different, *P* ≤ 0.05.

Table S2 Effect of salinity levels (0, 50, and 100 mM NaCl), ethanol application (no ethanol, with ethanol) and storage period (7 and 14 days) on water loss (%), color (*L*, *a^∗^*, *b^∗^* values), total phenolic content (mg GAE/g Fwt), antioxidants (mg trolox/g Fwt), carotenoids (mg/100 g Fw) and anthocyanins (mg cyn-3-glu/100 g Fw) on tagetes flowers during postharvest storage. ns, ^∗^, ^∗∗^, and ^∗∗∗^ indicate non-significant or significant differences at *P* ≤ 5%, 1%, and 0.1%, respectively, following two-way ANOVA.
