# Supplementary figures and images for: Effects of Salinity on Tagetes Growth, Physiology, and Shelf Life of Edible Flowers Stored in Passive Modified Atmosphere Packaging or Treated With Ethanol
Source: Front Plant Sci. 2018 Dec 10;9:1765. doi: 10.3389/fpls.2018.01765 (PMC6296340; doi:10.3389/fpls.2018.01765)

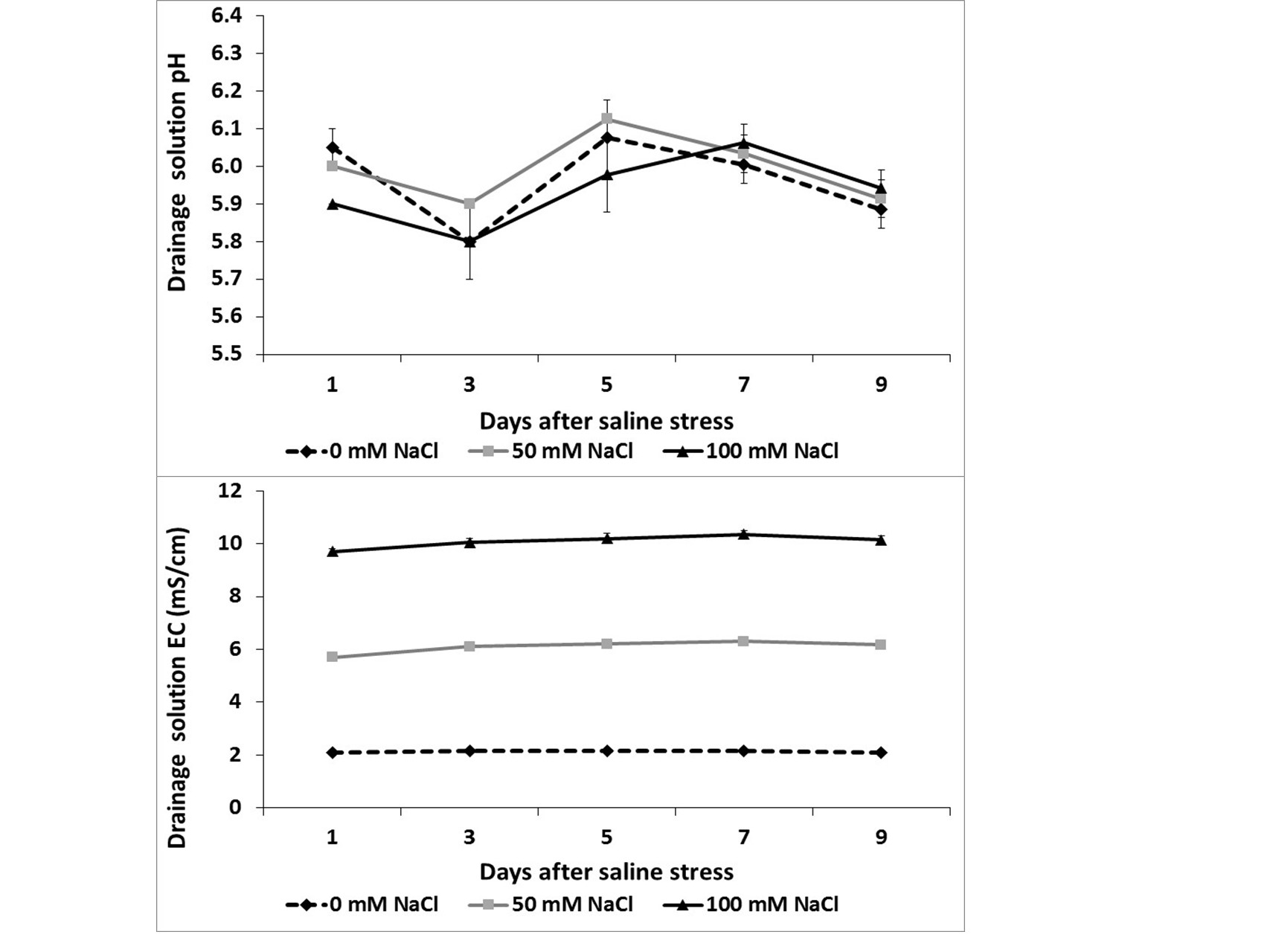

Supplement: Figure S1 — Fluctuation of drainage pH and EC (mS/cm) under different salinity levels (0, 50, and 100 mM NaCl) in hydroponically grown tagetes plants. [file Image_1.jpg]

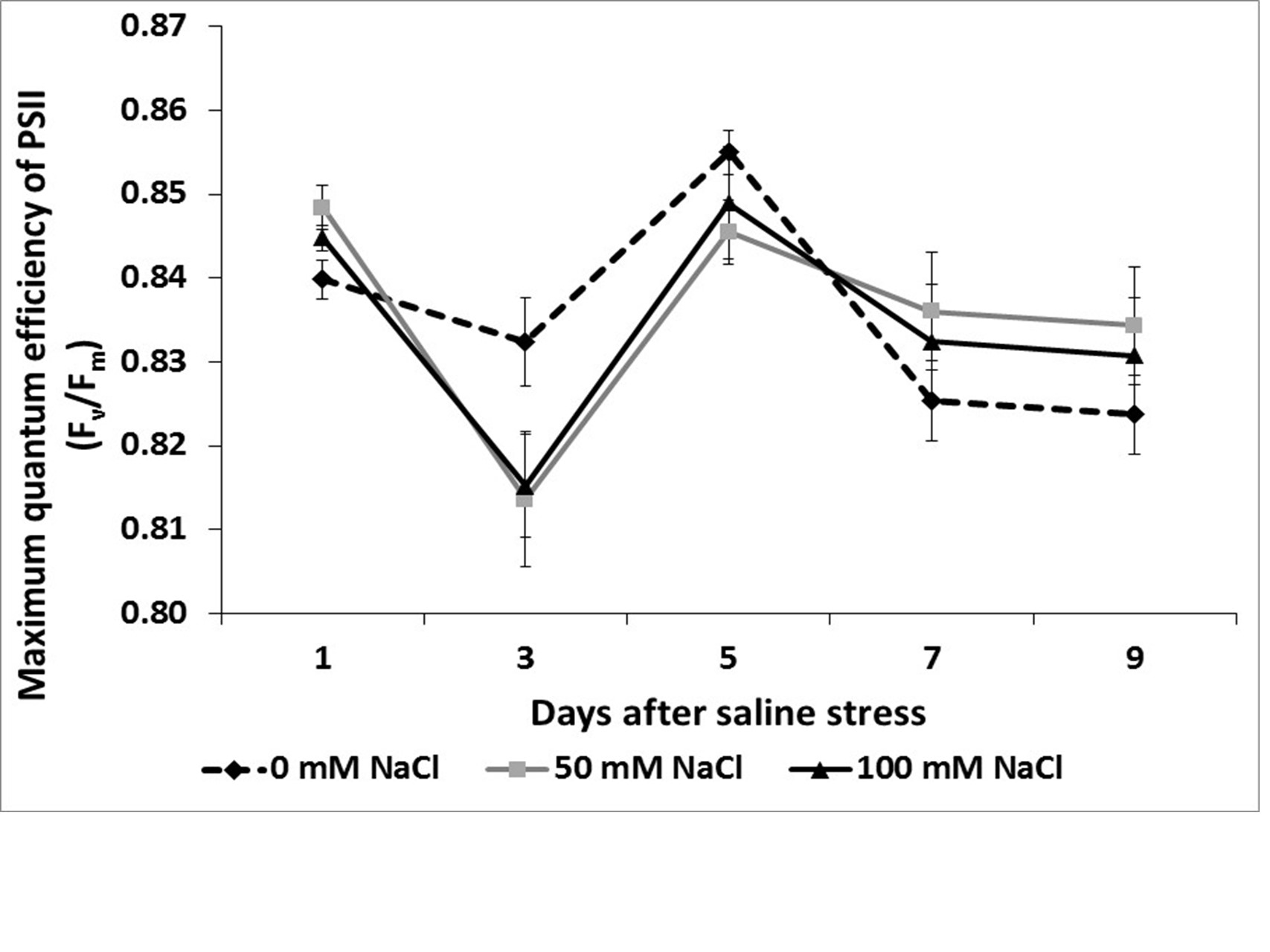

Supplement: Figure S2 — Effect of salinity levels (0, 50, and 100 mM NaCl) on tagetes maximum quantum efficiency of PSII (Fv/Fm) in plants grown hydroponically. [file Image_2.jpg]
