# Supplementary material for: Effects of Salinity on Tagetes Growth, Physiology, and Shelf Life of Edible Flowers Stored in Passive Modified Atmosphere Packaging or Treated With Ethanol
Source: Front Plant Sci. 2018 Dec 10;9:1765. doi: 10.3389/fpls.2018.01765 (PMC6296340; doi:10.3389/fpls.2018.01765)
Supplement: Table S1 — Effect of salinity levels (0, 50, and 100 mM NaCl) on tagetes flowers color values (L, a∗, b∗, Chroma) in plants grown hydroponically. YResults are expressed as means±SE (n = 6). Values in rows followed by the same letter are not significantly different, P ≤ 0.05. [file Table_1.docx]

**Supplementary Table 1S**. Effect of salinity levels (0-50-100 mM NaCl) on tagetes flowers color values (*L*, *a**, *b**, Chroma) in plants grown hydroponically.

|  | **0 mM NaCl** | **50 mM NaCl** | **100 mM NaCl** |
| --- | --- | --- | --- |
| **Colour L** | 55.75±5.07a ^Y^ | 54.62±3.43a | 56.63±5.36a |
| **Colour a*** | 43.20±1.80a | 41.55±0.84a | 39.59±2.75a |
| **Colour b*** | 87.67±8.01a | 85.06±5.40a | 85.53±7.82a |
| **Chroma** | 98.47±6.20a | 94.87±4.72a | 95.13±5.95a |

^Y^ Results are expressed as means±SE (n=6). Values in rows followed by the same letter are not significantly different, *P*<0.05.
