# Supplementary material for: Effects of Salinity on Tagetes Growth, Physiology, and Shelf Life of Edible Flowers Stored in Passive Modified Atmosphere Packaging or Treated With Ethanol
Source: Front Plant Sci. 2018 Dec 10;9:1765. doi: 10.3389/fpls.2018.01765 (PMC6296340; doi:10.3389/fpls.2018.01765)
Supplement: Table S2 — Effect of salinity levels (0, 50, and 100 mM NaCl), ethanol application (no ethanol, with ethanol) and storage period (7 and 14 days) on water loss (%), color (L, a∗, b∗ values), total phenolic content (mg GAE/g Fwt), antioxidants (mg trolox/g Fwt), carotenoids (mg/100 g Fw) and anthocyanins (mg cyn-3-glu/100 g Fw) on tagetes flowers during postharvest storage. ns, ∗, ∗∗, and ∗∗∗ indicate non-significant or significant differences at P ≤ 5%, 1%, and 0.1%, respectively, following two-way ANOVA. [file Table_2.docx]

**Supplementary Table 2S.** Effect of salinity levels (0-50-100 mM NaCl), ethanol application (no ethanol, with ethanol) amd storage period (7 days and 14 days) on water loss (%), colour (*L*, *a**, *b** values), total phenolic content (mg GAE/g Fwt), antioxidants (mg trolox/g Fwt), carotenoids (mg/100 g fw) and anthocyanins (mg cyn-3-glu/100 g fw) on tagetes flowers during postharvest storage.

| Two-way Anova | Water loss | L | a* | b* | Phenols | DPPH | FRAP | ABTS | Car | Anth |
| --- | --- | --- | --- | --- | --- | --- | --- | --- | --- | --- |
| Period (Per) | ns | ns | ns | ns | * | *** | ns | ns | ns | ns |
| Salinity (Sal) | * | ns | ns | ns | ns | ns | ns | ns | ns | ns |
| Ethanol (ET) | ns | ns | ns | ns | ns | ns | ns | ns | ** | *** |
| Per*Sal | ns | ns | ns | ns | ns | ns | * | ns | ns | ns |
| Per*ET | ns | ns | ns | ns | ns | ns | ns | ns | ns | ns |
| Sal*Et | ns | ns | ns | ns | ns | *** | ns | ns | ns | ns |
| Per*Sal*ET | ns | ns | ns | ns | ns | *** | ns | ns | ns | ns |

ns, *, **, and *** indicate non-significant or significant differences at *P*< 5%, 1% and 0.1%, respectively, following two-way ANOVA.
